# How Do Yeast Cells Contend with Prions?

**DOI:** 10.3390/ijms21134742

**Published:** 2020-07-03

**Authors:** Reed B. Wickner, Herman K. Edskes, Moonil Son, Songsong Wu, Madaleine Niznikiewicz

**Affiliations:** Laboratory of Biochemistry and Genetics, National Institute of Diabetes Digestive and Kidney Diseases, National Institutes of Health, Bethesda, MD 20892-0830, USA; hermane@niddk.nih.gov (H.K.E.); moonil.son@nih.gov (M.S.); wusong4225@gmail.com (S.W.); magdanizni@gmail.com (M.N.)

**Keywords:** prion, anti-prion, Btn2, Cur1, Hsp104, Upf, Ssb, Lug1, Siw14, amyloid

## Abstract

Infectious proteins (prions) include an array of human (mammalian) and yeast amyloid diseases in which a protein or peptide forms a linear β-sheet-rich filament, at least one functional amyloid prion, and two functional infectious proteins unrelated to amyloid. In *Saccharomyces cerevisiae,* at least eight anti-prion systems deal with pathogenic amyloid yeast prions by (1) blocking their generation (Ssb1,2, Ssz1, Zuo1), (2) curing most variants as they arise (Btn2, Cur1, Hsp104, Upf1,2,3, Siw14), and (3) limiting the pathogenicity of variants that do arise and propagate (Sis1, Lug1). Known mechanisms include facilitating proper folding of the prion protein (Ssb1,2, Ssz1, Zuo1), producing highly asymmetric segregation of prion filaments in mitosis (Btn2, Hsp104), competing with the amyloid filaments for prion protein monomers (Upf1,2,3), and regulation of levels of inositol polyphosphates (Siw14). It is hoped that the discovery of yeast anti-prion systems and elucidation of their mechanisms will facilitate finding analogous or homologous systems in humans, whose manipulation may be useful in treatment.

## 1. Introduction

The bovine spongiform encephalopathy (“Mad Cow Disease”) epidemic in the UK brought to public attention the rare, uniformly fatal infectious neuropathies based on amyloid formation by a normal cell surface protein called PrP. However, current work suggests that many of the common human amyloid-based neural degenerative diseases, such as Alzheimer disease, Parkinson disease, and amyotrophic lateral sclerosis, as well as type 2 (late onset) diabetes, have many aspects in common with the PrP-based diseases, including frank infectivity [1,2,3,4]. The discovery of prions in *Saccharomyces cerevisiae* enabled the acceleration of understanding of such diseases, and has recently led to the detection of cellular systems that may be viewed as innate immunity to prions.

The [URE3] and [PSI+] non-Mendelian genetic elements of *S. cerevisiae* [5,6] were found to be prions of Ure2p and Sup35p, respectively, based on their outré genetic properties [7,8,9]. Ure2p is a negative regulator of transcription of genes important for the utilization of poor nitrogen sources. In the presence of a good nitrogen source, such as ammonia or glutamine, Ure2p shuts off expression of genes, including *DAL5*, encoding the allantoate transporter, that are needed for full utilization of poor nitrogen sources [10]. By sequestering Ure2p in amyloid filaments [7,11,12,13,14], the [URE3] prion derepresses *DAL5,* detected experimentally as expression of a *DAL5:ADE2* fusion gene [14,15] or through the Dal5p-facilitated uptake of the uracil intermediate ureidosuccinate in the presence of a good nitrogen source. 

Sup35p is a subunit of the translation termination factor [16,17], and the sequestration of Sup35p in the [PSI+] amyloid filaments elevates the frequency with which premature nonsense codons are read through [7,18,19,20,21,22]. 

[PIN+] (for [**P**SI]-**in**ducibility [23,24,25]) is a prion of Rnq1p, a protein of unknown function. [PIN+] was detected by its ability to rarely seed the formation of the [PSI+] prion [23]. Several other yeast and fungal prions have now been found (Table 1), including the functional prion [Het-s] of the filamentous fungus *Podospora anserina*, which controls heterokaryon incompatibility (like an HLA locus) in this organism [26,27].

## 2. Prion Variants

A given prion protein sequence can be the basis of many distinct prions (called prion variants or prion strains), differing in the phenotype they confer on their yeast or mammalian host. In yeast, prion variants may differ in the intensity of the prion phenotype (strong vs. weak), the stability of the prion propagation, sensitivity to overproduction or deficiency of some cellular component, ability to propagate from cells with one prion protein sequence to another (e.g., the species barrier), and/or the toxicity of the prion to the host ([23,38,39,40,41,42,43,44,45,46,47,48], reviewed in [49,50]). Most prion variants are remarkably stable in their properties, but a number of prion “mutations” have been described [42,43,47,48,51,52]. Even under non-selective conditions, distinct prion variants in the same host also show segregation during mitosis [52]. At least some (and presumably all) variant differences are due to differences in the detailed folding/structure of the amyloids, although their amino acid sequences are all the same [53]. The fact that these structures are all self-propagating was, at first, a reason why some were skeptical that there could be such a thing as a prion, but structural studies of infectious amyloids of yeast prion proteins later led to an understanding of how a protein could template its own architecture (see below).

## 3. Structures of Yeast/Fungal Prion Amyloids: Explanation of Templating

**Figure 1:**
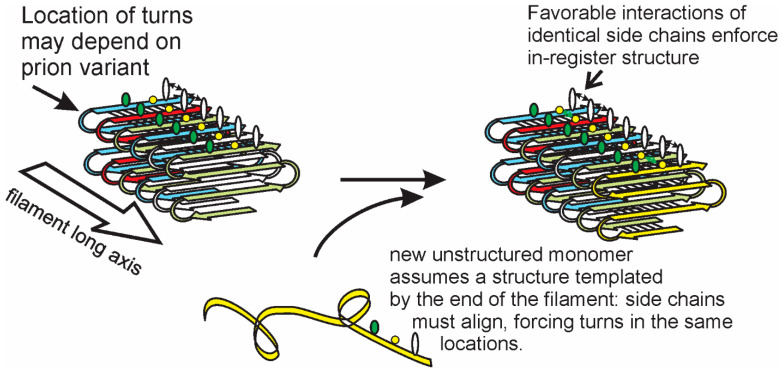
The in-register folded parallel β-sheet architecture of infectious amyloid of Ure2p, Sup35p, and Rnq1p prion domains suggests a mechanism of conformational templating that can explain how different prion variants of the same protein can each faithfully propagate.

Solid-state NMR studies, combined with information from electron microscopy and support from electron spin resonance, has led to the in-register parallel folded β-sheet model for the yeast prions [URE3], [PSI+], and [PIN+] (Figure 1, reviewed in [9,54]). The unique characteristic of this architecture is that each amino acid residue in the β-sheet part forms a line of identical residues along the long axis of the filament, with adjacent residues in this line separated by the distance between the strands of a β-sheet, namely, ~4.8 angstroms. Using amyloid in which a single residue is labeled with one ^13^C atom (usually the alpha carbonyl carbon) in each molecule, the solid-state NMR experiments measure exactly this distance, an approach first developed by Benzinger et al. [55] using a fragment of Aβ. This was the result obtained in various experiments for infectious filaments of the prion domains of Ure2p, Sup35p, and Rnq1p [56,57,58,59,60,61] and supported by electron spin resonance experiments [62,63] and measurements of filament mass per unit length [64,65,66].

Actually, this prion architecture was first proposed based on the observation that shuffling the amino acid sequence of the prion domains of Ure2p or Sup35p did not prevent these proteins from forming prions [67,68,69]. This startling result, combined with the sequence specificity for prion propagation (see above), could be explained by the in-register parallel architecture, but not by anti-parallel or β-helix models [69].

If the yeast prion amyloid filament consisted of a flat unfolded β-sheet, it would be a ribbon over 20-nm wide. In fact, the filaments are only 3–4 nm in diameter, proving that the β-sheet must have several folds [13,19] (Figure 1), a result confirmed by cross-linking studies [70]. It has been suggested that the locations of these folds may differ among prion variants [9,71] (Figure 1), and solid-state NMR and cross-linking studies support this suggestion [60,70]. Moreover, it is expected that the same favorable H-bonding or hydrophobic interactions between identical amino acid residue side chains that keep the structure in-register will guide a new molecule, joining the end of the filament to assume the same structure, with the folds in the same locations as the last monomer on the end of the filament [9,71]. This provides a templating mechanism allowing a protein to template its own conformation, in analogy to the sequence templating by replicating DNA or RNA [9,71] (Figure 1, modified from [9]).

## 4. Biology of Yeast/Fungal Prions

Most isolates of the [PSI+] or [URE3] prions are toxic or even lethal to their hosts [46], suggesting that prion-forming ability is not an advantageous trait. Most laboratory studies use variants of these prions that are mild in their effects under the usual growth conditions, although all [URE3] variants slow cell growth (e.g., [72]), and [PSI+] makes cells slow to emerge from stationary phase [73].

Many detrimental infectious agents are common in the wild because infection may outrun the elimination of the host by disease. For example, the lethal prion disease of elk and deer, Chronic Wasting Disease, has become quite common in many areas of the United States. An infectious condition that had a net benefit to the host would certainly spread rapidly in the wild. Because yeast prions arise spontaneously at frequencies of 10^−5^ or 10^−6^, the yeast population on one grape would likely have a cell with a prion. Benefit to the host, infectivity, and spontaneous occurrence would all be working to make such a prion commonplace in wild populations. Thus, if a prion is rare in wild isolates, it must be detrimental. Indeed, [PSI+] and [URE3] were not found in 70 wild isolates from a wide variety of sources, and [PIN+] was found in only a minority, implying that all three prions are diseases [74]. Both prions and the two micron DNA plasmid of yeast spread by mating/meiosis, and two micron DNA has been shown to slow yeast growth by 1 to 3% [75,76,77]. Nonetheless, this plasmid was found in a majority of the 70 wild isolates, indicating that these prions have a greater than 1–3% detrimental effect in the wild [78]. Note that the [Het-s] prion, responsible for an aspect of heterokaryon incompatibility in *Podospora anserina* (a normal function), is indeed found in 95% of wild strains [79], as expected for a beneficial prion. The infectious amyloid of the [Het-s] prion is a β-helix, a structure which assembles in a unique manner to form a single structure, resulting in constituting only a single prion variant for [Het-s] [27,80,81], as expected for a functional prion.

We note here a slight confusion of nomenclature that has arisen. We use “[PSI+] toxicity” (or that of [URE3] or other prions) to refer to the detriment to growth or survival of an otherwise normal cell carrying a particular prion variant [46]. This prion toxicity can be due to the functional deficiency of an essential protein because most is taken up by the filaments (as in a majority of [PSI+] variants) or by some toxic action of the prion amyloid that cannot be accounted for by lack of the normal protein (as in the case of [URE3]) [46]. In contrast, the same expression is used in reference to the lethality of [PSI+] resulting from overproduction of Sup35p or Sup35NM, a very distinct phenomenon shown clearly by Vishveshwara et al. to be due to the elevated amount of amyloid-sequestering of Sup45p or full-length Sup35p, respectively [82]. Another example of this latter type of “prion toxicity” is the sequestration of Spc42p, an essential spindle pole body component, by Rnq1p amyloid filaments in a [PIN+] cell in which Rnq1p is overproduced [83]. Our use of “prion toxicity” speaks to the issue of whether specific prions are advantageous (like [Het-s]) or detrimental (like [PSI+] and [URE3]). The other phenomena involving prion protein overproduction have revealed interesting mechanisms by which a prion might harm the cell, but do not directly address the benefit–detriment issue.

## 5. Prions Evolve on Two Levels

We think of evolution as operating on the DNA sequence of genes to select those which give the cell/organism the most advantageous phenotype. However, sequence differences in the prion domain often block the ability of a prion originating in one cell to propagate in another. Such differences can reflect interspecies differences (see references above) or artificially constructed differences [84]. Intraspecies differences in the prion domain of Sup35p also produce a barrier to transmission [47]. In this case, a sequence change in the prion domain may not improve the non-prion function of the protein, but will be selected because it prevents infection with the detrimental [PSI+] prion. Of course, the prion domains are also constrained by the normal functions of these proteins. The Ure2p prion domain is necessary for the stability against degradation of the protein [85], and the prion domain of Sup35p is required for proper mRNA turnover [86] and recovery from stationary phase by promoting liquid phase separation [73].

But prions are molded by evolution on a second level, because they are heritable, acting as (non-chromosomal) genes, and template their own conformation, but with some occasional errors. The templating errors produce variability in prion properties, which are then selected by evolution. The most toxic prions are rapidly lost as cells in the population with less toxic variants quickly outgrow those with the original more lethal variant [46]. Variants of [URE3] sensitive to normal levels of the anti-prion proteins Btn2p and Cur1p (see below) occasionally mutate into relatively insensitive variants, which are then selected for in normal cells [48]. This is the second level of evolutionary selection that is unique to prions. Each level affects the other. The amino acid sequence of the prion domain affects which prion variants can arise [84], in addition to determining the non-prion function. If a specific prion variant is particularly toxic, there will be selection for prion domain sequence changes that are incompatible with that variant (reviewed in [87]).

## 6. Chaperones and Prions 

The duplication of amyloid-based prions consists of the splitting of filaments to make new growing ends (propagons/seeds). This process is carried out by the Hsp104/Hsp70 (Ssa1,2)/Hsp40 (Sis1/Swa2)/NEF (Fes1, Sse1) chaperone machinery [88,89,90,91,92,93,94,95,96,97]. Biochemical data indicate that the Hsp70/Hsp40 combination brings together Hsp104 and a target in the amyloid filament, and Hsp104 draws out a peptide chain from the filament through a hole in the center of its hexamer, thereby breaking the filament (one monomer is one layer of the filament) and allowing the withdrawn monomer to refold [98,99,100,101]. Several modulators of the Hsp70 ATPase cycle, by affecting the opening and closing of the chaperone to substrate, affect the efficiency of Hsp70 cooperation with Hsp104 and the propagon generation reaction ([102], reviewed by [103]).

Overproduction of Hsp104 cures the [PSI+] prion [88,104], an activity that is not due to over-cleavage of filaments [92], but is rather a distinct activity, involving the N-terminal part of the Hsp104 molecule [99]. The Hsp104 overproduction curing activity also involves Hsp70 [99] as well as Hsp90s and their co-chaperones [105,106]. A region of the Sup35M domain has been identified as the target of the Hsp104 overproduction [PSI+]-curing activity [107]. As discussed below, the Hsp104 overproduction curing activity culls a large fraction of [PSI+] variants that arise in the absence of that activity (and presumably in its presence as well). Another physiologic role of this activity is curing of [PSI+] as a result of a transient temperature elevation, such as must often occur in the wild [108,109]. This destabilization of [PSI+] requires the Hsp104 overproduction–curing activity, as shown by its loss in the N-terminal mutants [110]. Interestingly, the heat-pulse curing requires Sir2p, and the Hsp104 overproduction curing activity is slower without Sir2p [110]. Sir2p is an NAD+-dependent histone deacetylase originally identified as a repressor of information at the silent mating type loci at the extremes of chromosome III. Sir2p is necessary for the asymmetric segregation in mitosis of damaged (e.g., oxidized) proteins [111], a process that is blocked by nicotinamide, an inhibitor of the histone deacetylase [112]. However, nicotinamide did not inhibit the heat-pulse curing of [PSI+] [110]. Nonetheless, the finding that that the damaged protein system [111] works in prion curing [110] is an important advancement.

Hsp90s and their various co-factors affect prions in another (unexpected) way. Lancaster et al. found that *sba1*Δ made a strong variant of [PIN+] (able to efficiently prime [PSI+] formation) become a weak variant, while *hsc82*Δ, *aha1*Δ, *cpr6, cpr7*Δ, and *tah1*Δ had the opposite effect [113]. These were not merely changes in prion phenotype, but [PIN+] remained changed on transfer of the prion to a wild-type host. Each of these genes encodes an Hsp90 (*HSC82*) or an Hsp90 co-chaperone.

Hsp90s and their co-chaperones have also been implicated in the [URE3] prion propagation process. Hsp90s interact with TPR-containing chaperones through the C-terminal sequence MEEVD. Kumar et al. found that deleting this sequence resulted in destabilization of [URE3] (but not [PSI+]) [114]. Among the Hsp90 co-chaperones interacting with this sequence, Cpr7p was specifically required to stabilize [URE3] propagation and was shown to directly interact with Ure2p [114]. Swa2p, an Hsp40, is also needed for [URE3] propagation, and appears to interact with Hsp70 (Ssa) through its J-domain and with Hsp90 through the Swa2p TPR repeat domain in carrying out this activity [115].

Among Hsp40s, Sis1p (the only essential Hsp40 [116]) is required by [PSI+], [URE3], [PIN+], and [SWI+] [94,117,118], while Swa2 is specifically required for [URE3] [97]. The specificity appears to reside in the TPR domain of Swa2. Sis1p also has a role in the Hsp104 overproduction curing of [PSI+] [119,120] and, for curing some strong [PSI+] variants, Apj1p is also necessary [120].

## 7. Anti-Prion Systems

**Figure 2:**
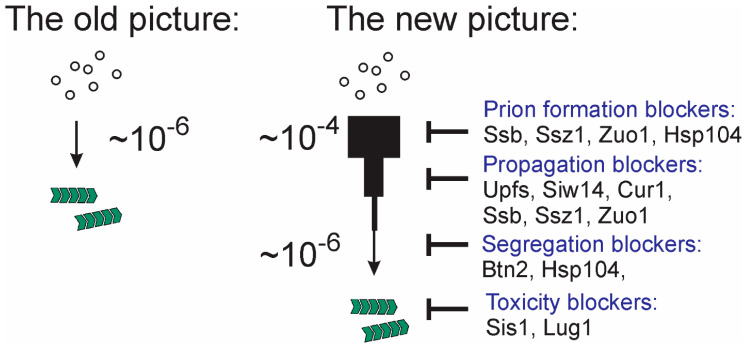
The existence and properties of the many yeast anti-prion systems suggest that prion formation is not a trickle, but rather a tidal wave that is attenuated by many systems at several levels. The anti-prion roles of Siw14 and Cur1 are not yet clear, but are listed here as blocking prion “propagation” in the broad sense.

From the pathogenic nature of the [URE3] and [PSI+] prions, one would expect cells to have systems to prevent prion formation, to cure prions that do form, and to limit the damage done by any prions surviving such measures. Each of these expectations have been met. We will see that the current concept of prions now differs considerably from that of the fairly recent past. It was formerly reasonable to suppose that prions arose at a mercifully low frequency (~10^−6^) and that while some were inherently unstable, they would propagate and have their effects, including lethality. However, the current picture (Figure 2) is that prions arise at a much higher frequency (not yet measured, but shown as 10^−4^ in Figure 2), with rapid culling of nearly all prions, leaving only a few able to propagate.

### 7.1. Ribosome-Associated Complex

Ssb1p and Ssb2p are nearly identical Hsp70s associated with the ribosome where, as part of the RAC (ribosome associated complex) with Zuo1p (an Hsp40) and Ssz1 (another Hsp70), they are responsible for proper folding of nascent proteins [121,122]. For example, *ssb, ssz1,* and *zuo1* mutants accumulate aggregates of many proteins [123]. Chernoff has shown that *ssb1*Δ *ssb2*Δ strains generate [PSI+] prions at a 10-fold elevated frequency [124,125]. Restoring the *SSB1* gene in *ssb1*Δ *ssb2*Δ strains that have become [PSI+] did not cure the prion, indicating that the mutant was not allowing different prion variants to propagate, but was having an effect on prion generation [125]. In *ssz1*Δ and *zuo1*Δ mutants, [PSI+] generation is similarly much more frequent [126,127]. Although these RAC components do not seem to act in lowering [PSI+] generation by blocking prion propagation, overproduction of Ssb1p stimulates curing of a weak [PSI+] variant by overproduced Hsp104 [125]. The *ssz1*Δ and *zuo1*Δ mutations, which result in Ssbs no longer being ribosome-associated, have the same effect on Hsp104-overproduction curing [126], and overproduced Ssb1p can cure an artificial prion [128] or a weak [PSI+] prion [129] even without Hsp104 overproduction. Thus, there is substantial evidence that overproduced, soluble Ssb1p can be prion-curing.

***Btn2p sequesters prion aggregates, which cures many progeny cells.*** In a screen for high-copy genes that cure [URE3], the paralogous *BTN2* and *CUR1* were each isolated [130]. Prion aggregates of Ure2p are normally scattered about the cytoplasm [12], but Btn2p was found to gather these aggregates to one place in the cell, with Btn2-RFP co-localizing with Ure2p-GFP [130,131]. It was proposed that sequestration of Ure2p amyloid at one locus in the cell results in frequent production of progeny cells that have no aggregates and, so, are cured of the prion [130]. Overproduced Btn2p can also cure an artificial prion, namely an Nrp1-Sup35C fusion protein [132], and co-localizes with several other non-prion aggregates of misfolded foreign proteins [130,132,133,134], so it is not exclusively directed at prions. Btn2p shows a modest, but significant, homology with human HOOK1 [135], an adapter/activator for dynein connecting a motor protein with its cargo, signaling endosomes in this case [136]. Btn2p overproduction curing requires Hsp42, and Hsp42 overproduction by itself also cures [URE3] [48] and collects non-prion aggregates as well [137]. However, another study reports that overproduced Btn2p and Hsp42 collect non-prion aggregates in separate compartments: Btn2p in the nucleus and Hsp42 in the cytoplasm [138]. Thus, while the exact mechanism is not yet clear, Btn2p clearly cures prions (and clears other aggregates) by sequestration.

### 7.2. Cur1p Cures [URE3] without Visibly Collecting Ure2p Amyloid Aggregates

Although isolated in the same screen and having substantial similarity with Btn2p, Cur1p does not visibly sequester Ure2p amyloid aggregates and probably cures by a different mechanism [130]. Sis1p is an Hsp40 that is necessary for the propagation of [URE3], [PSI+], and [PIN+] through its involvement in the Hsp104-Hsp70-Hsp40 filament-splitting process [94,139]. It was proposed that Cur1 and Btn2 cure by sequestering Sis1p in the nucleus because overproduced Sis1p blocked curing by overproduced Btn2p or Cur1p [132,140], however other evidence (see below) brings this view into question.

### 7.3. Normal Levels of Btn2p and Cur1p Cure Most [URE3] Variants Arising in Their Absence

To determine whether Btn2p or Cur1p could cure prions without being overexpressed, an array of [URE3] variants was selected in a *btn2*Δ *cur1*Δ strain, and then prion stability was examined after restoring normal levels of both proteins by either mating with a wild-type [ure-o] strain or transformation with single-copy plasmids carrying one gene or the other or both [48]. Surprisingly, nearly all the [URE3] variants arising in the *btn2*Δ *cur1*Δ strain were cured by restoring normal levels of Btn2p and/or Cur1p [48]. In both this normal level curing and the overexpression curing Btn2p and Cur1p, each acted independently of the other, although both required Hsp42 for the curing [48]. In *btn2*Δ *cur1*Δ strains, the frequency of [URE3] arising spontaneously is about five-fold higher than for a normal strain, and over 90% of the variants arising are cured upon restoring the normal amounts of either or both proteins [48]. The [URE3] variants that are cured by normal levels of Btn2 and Cur1 have a much lower seed number than do those only curable by overproduction of one of the proteins [48], consistent with the sequestration model of Btn2p action [130].

The protein abundance database (https://pax-db.org/), summarizing a large number of studies, gives the abundance of Cur1p as 1.4 ppm (~140 molecules/cell) and Btn2p as 6.1 ppm (~300 molecules/cell), while Sis1p is present at about 5 × 10^4^ molecules/cell. The fact that Btn2p and Cur1p are present in normal cells at levels of only 100- to 300-fold below that of Sis1p, and yet can cure most [URE3] variants at that level, suggests that they are not doing so by sequestering Sis1p. On the contrary, it is possible that overproduction of Sis1p sequesters Btn2p and Cur1p, preventing their action. Moreover, the Sis1p sequestration model does not explain the co-localization of Btn2p with Ure2p aggregates in cells undergoing curing [130,131]. In addition, deletion of the putative Btn2p nuclear localization sequence did not abrogate curing of [URE3] [130]. Nonetheless, it remains possible that *overproduced* Cur1p acts in this way.

The fact that normal levels of Btn2p or Cur1p could each cure most [URE3] prion variants arising in their absence casts a new light on the dynamics of the interactions of prions and their hosts. It showed that there is an array of prion variants that had not yet been studied because they could not propagate in wild strains. Moreover, the rather high efficiency of these anti-prion systems suggests that yeast is not entirely delighted to be infected by prions.

### 7.4. Hsp104 Curing Activity Works at Normal Chaperone Levels

As discussed above, Hsp104 has both a prion-promoting activity (filament breaking) and a prion-curing activity (asymmetric segregation). The *hsp104^T160M^* allele is unable to cure [PSI+], even if overproduced, but can support normal propagation of either [PSI+] or [URE3] [99]. Following the approach used in studying Btn2 and Cur1, [PSI+] isolates were obtained in this mutant, and at least half of the isolates were lost upon transfer to a wild-type host (without any protein overexpression) but not if transferred to another host with the same *hsp104^T160M^* mutation [141]. The frequency of spontaneous [PSI+] was elevated about 13-fold in this mutant, again implying that Hsp104 is normally eliminating a large fraction of the [PSI+] prion variants that arise in the cell.

### 7.5. Siw14p, Inositol Polyphosphates, and [PSI+] Prion Propagation

A general screen of the yeast knockout collection for anti-prion components found that about half of [PSI+] variants arising in *siw14*Δ strains were eliminated on their return to a normal host [142]. Siw14p is a pyrophosphatase specific for 5-pyrophosphoryl-inositol-pentakisphosphate (PP-IP_5_) [143], one of the soluble inositol polyphosphate signaling molecules involved in an array of cellular processes [144]. In *siw14*Δ strains, PP-IP_5_ is present at a six-fold higher level than that found in wild-type cells [143]. It was inferred that some [PSI+] variants required elevated levels of PP-IP_5_ in order to propagate [142]. PP-IP_5_ is involved in an environmental stress response (ESR) reaction in which response to heat, oxidation, or osmotic stress requires 5PP-IP_5_, 1PP-IP_5_ and 1,5PP-IP_4_, a response eliminated in *kcs1*Δ *vip1*Δ strains [145,146]. However, the [PSI+] variants dependent on PP-IPs are not lost in *kcs1*Δ *vip1*Δ strains, indicating that the ESR system is not responsible for the prion effects [142].

Arg82p is a kinase early in the inositol polyphosphate pathway, which is necessary for the biosynthesis of most of these molecules. It was found that in *arg82*Δ cells, most variants of [PSI+] were lost [142]. Examination of mutants in the biosynthetic pathway showed that 5PP-IP_4_, IP_6,_ or 5PP-IP_5_ were each sufficient for the propagation of the few [PSI+] variants that were tested. Although IP_6_ and 5PP-IP_5_ have been shown to bind to a variety of cellular proteins, including some known to affect prion propagation (Ssbs, Sse1p, Hsp26) [147], the specific target molecule responsible for the effects on prions remains to be determined.

### 7.6. Nonsense-Mediated mRNA Decay Factors Upf1,2,3 Cure some [PSI+] Variants

The same screen detecting *siw14*Δ and the [PSI+] variants requiring elevated 5PP-IP_5_ levels showed that in *upf1*Δ and *upf3*Δ mutants, most [PSI+] variants arising were cured if normal levels of these proteins were restored [148]. Upf1, Upf2, and Upf3 form a complex on the ribosome in association with Sup35p that carries out nonsense-mediated decay (reviewed by [149]). An mRNA with a premature termination codon is degraded more rapidly than the same mRNA with an intact open reading frame. It was shown that the interaction of Upf1, Upf2, and Upf3 with Sup35p and with each other is important for blocking prion formation or curing the Upf-hypersensitive prions formed in their absence. The efficiency of nonsense-mediated decay is not critical, as long as the complex is formed [148]. Sub-stochiometric concentrations of Upf1p are sufficient to block amyloid formation by Sup35p in vitro, and Upf1p binds to the Sup35p prion amyloid filaments in vivo [148] and in vitro [150], suggesting that the Upf proteins may bind to filament ends, blocking their elongation. Alternatively, the normal Upf complex with Sup35p may compete for monomer such that many prion variant amyloid filaments cannot elongate. However, of most interest is the general notion that normal protein–protein interactions can prevent prion formation or cure prions that have formed.

### 7.7. Sis1p Prevents Toxicity of [PSI+] Variants that Are Mild in Normal Cells

Sis1p is an essential Hsp40 family member, but a detailed dissection of its domains has shown that constructs sufficient for cell viability in the absence of [PSI+] are insufficient to protect cells from the toxicity of strong variants of [PSI+] [119,151]. Cells expressing such Sis1p fragments, lacking the G/F domain and part of the C-terminal domain, can support growth and propagate weak [PSI+] variants, but are unable to maintain the [PIN+] prion.

### 7.8. Lug1p Lets [URE3] Grow

In a screen for genes that could be targeted by the *Hermes* transposon if they were [ure-o] but not if they carried the [URE3] prion, the most prominent hit was *YLR352w,* which was then named *LUG1* [152]. A *lug1*Δ *ure2*Δ strain shows the same growth defects as a *lug1*Δ [URE3] strain, so Lug1p is protecting against lack of Ure2p function, not the presence of Ure2p amyloid. Lug1p is the substrate-specifying subunit of a cullin-type or “SCF” E3 ubiquitin ligase [153]. Such E3s have four constant subunits and a fifth subunit with multiple alternative isoforms (20 in yeast) identified by a conserved “F-box” sequence, each isoform specifying the proteins to be modified by ubiquitin [154]. In this case, the targets of Lug1p are not yet known [153]. The growth defect of *lug1*Δ [URE3] strains, nearly complete on glycerol but still noticeable on glucose, is suppressed by overexpression of *HAP4,* consistent with a relation to carbon catabolism [152]. A *ure2*Δ mutation eliminates nitrogen catabolite repression (as does [URE3]). Paradoxically, mutation of *GLN1* that eliminates nitrogen catabolite repression also suppresses the lethality of *lug1*Δ *ure2*Δ [152]. Further work will evidently be needed to understand the mechanisms involved. Interestingly, alleles of the E3 ubiquitin ligases, *HECTD2* and *PARKIN,* are associated with susceptibility to PrP-based prion disease and Parkinson disease [155,156], an interesting parallel with our findings.

The *Hermes* screen also showed that several chaperones and co-chaperones not previously found needed for [URE3] propagation were disrupted less frequently in [URE3] cells than in [ure-o] cells [152]. These include *HSP82* and *HSC82* (the Hsp90 genes), *YDJ1* and *CAJ1* (Hsp40s), the Hsp90 co-chaperones *STI1* and *SBA1,* the nucleotide exchange factor *FES1, SSB1* and *SSB2* (ribosome-associated Hsp70s), and *HSP26* and *HSP42* (‘small’ Hsps). Whether the proteins encoded by these genes help prevent [URE3] toxicity or have subtle roles in [URE3] propagation is not yet clear.

## 8. Perspectives and Prospects

The existence of multiple anti-prion systems confirms that the yeast cell does not view these prions as ‘good’, a conclusion already secure based on other information. Of course, this does not mean that there are no ‘good prions’, as the [Het-s] prion of *Podospora* has amply shown. Anti-prion systems are not necessarily active only on prions. Btn2p, with its partner Hsp42, sequesters various non-prion, non-amyloid aggregates [132,133,137]. Hsp104 disaggregates heat-denatured proteins and is involved in retention of non-amyloid damaged proteins in the mother cell [157]. Ssbs, Ssz1, and Zuo1 are involved in the proper folding of nearly all nascent proteins. Swi14 affects regulation of the environmental stress response system [145], although it is not clear yet how inositol polyphosphates affect prions.

The anti-prion systems found in yeast may have either homologs or analogs in human cells. A protein interacting with a potentially prion-forming protein should, like Upf proteins, compete with amyloid filaments for the supply of the normal form of the protein. There are human sequestering organelles (e.g., the aggresome [158]) which may have functions similar to that of Btn2p, Hsp42, and Hsp104 in yeast. Although this kind of study is more difficult in mammals, evidence for repression of prion propagation by normal levels of Hsp70 in mice has been reported [159]. That is, in HSP70-deficient mice, prion disease proceeds more rapidly. It might be impractical to measure whether such mice generate prions more frequently or if some prion variants arising in such animals would be cured by replacement of normal levels of Hsp70.

It is hoped that understanding of these systems will facilitate the development of treatments for human amyloid diseases.

Among interesting questions are:

>> What exactly do Hsp90s and their co-chaperones do to/for prions?
>> What are the relations among the various anti-prion components? Are they co-operating in some systematic way or are they just different systems that happen to have similar effects?
>> Which anti-prion systems are inducible (like Hsp104) and which are constitutive?
>> Are there mammalian anti-prion systems?
>> What are the detailed mechanisms of the yeast anti-prion systems? This applies to all of the systems discussed here, but particularly Lug1p, inositol polyphosphates, and Cur1p.

In pursuing the yeast anti-prion systems, we are continuously looking at parallels in immune systems directed against viruses and bacteria, and the many DNA repair systems with their specificity for type of lesion. The widespread occurrence of human amyloidosis and the already great variety of detected yeast anti-prion systems make us certain that similar systems will be found in mammals.

## Figures and Tables

**Table 1:** Prions of yeast and fungi.

Prion	Prion Protein	Prion Phenotype	Normal Protein Function	Reference
[URE3]	Ure2p	Derepressed genes for using poor N-sources in presence of a good N-source; slow growth	repression of genes for utilizing poor N-sources in presence of a good N-source	[7]
[PSI+]	Sup35p	Readthrough of termination codons; slow growth; death	translation termination	[7]
[PIN+] or [RNQ+]	Rnq1p	Rare generation (by cross-seeding) of [PSI+] or [URE3]	none known	[28]
[OCT+]	Cyc8p	Slow growth; impaired mating and sporulatiion	transcription repressor subunit	[29]
[SWI+]	Swi1p	Poor growth on raffinose, galactose or glycerol	chromatin remodeling subunit	[30]
[MOT+]	Mot3p	Inappropriate derepression of anaerobic genes; colony polymorphisms	transcription regulator	[31]
[MOD+]	Mod5p	Partial azole-resistance; slow growth	tRNA isopentenyltransferase	[32]
[BETA]	Prb1p	Active protease B (non-amyloid prion) *	Active protease B (this is a functional prion)	[33]
[SMAUG+]	Vts1p	Increased mRNA decay *	stimulates mRNA degradation	[34]
[Het-s]	HET-s	Heterokaryon incompatibility	Heterokaryon incompatibility (this is a functional prion)	[26]
[LSB+]	Lsb2p	Transient Pin activity ([PSI+] prion generation)	Inhibitor of actin filament nucleation	[35]

The intensity of prion phenotypes depends on the prion variant. All but [BETA] and [SMAUG] are amyloid-based prions. [Het-s] is a prion of the filamentous fungus *Podospora anserina,* and the others are prions of *S. cerevisiae.* The [Het-s] prion can propagate in *S. cerevisiae* [36], and there is evidence that the tumor-suppressor protein p53 can act as a prion in yeast [37]. [LSB+], a prion induced by thermal stress, propagates indefinitely, but substantially more slowly than the rate that the cells divide, so only a small minority of subclones of an [LSB+] clone have the prion [35]. These prions are all amyloid-based except (*) [BETA] and [SMAUG+].
